# Synthetic and Natural Antifungals—Desirable and Hazardous Effects

**DOI:** 10.3390/ijms23179608

**Published:** 2022-08-25

**Authors:** Dejan Stojković, Marija Ivanov, Ana Ćirić

**Affiliations:** Department of Plant Physiology, Institute for Biological Research “Siniša Stanković”—National Institute of Republic of Serbia, University of Belgrade, Bulevar despota Stefana 142, 11000 Belgrade, Serbia

The increasing incidence of patients struggling with fungal infections, along with high losses in the production of different foods/crops due to fungal diseases presents a significant burden to healthcare, agronomy, and economies worldwide. There is an array of antifungals available on the market, but they are often not efficient enough, and their long-term application is associated with the rise in antifungal-resistant strains. Recently, researchers have focused on the development of novel antifungals, especially those with fewer side effects, higher efficacy, and novel mechanisms of action for avoiding resistance. Moreover, novel antifungal strategies, innovative methods of drug delivery, and synergistic approaches have also been explored. This Special Issue aims to present research on novel antifungal agents, with an emphasis on their antifungal mechanisms. Both human and plant fungal infections are targeted in the research published in this Special Issue, and an overview of antifungals of synthetic and natural origin is presented.

Potential novel antifungals for the treatment of human infections, mainly those caused by *Candida* species, have been explored thoroughly. Ivanov et al. [1] explored camphor and eucalyptol. Camphor exhibited promising antifungal potential (minimal inhibitory concentration, MIC 0.125–0.35 mg/mL). On the other hand, eucalyptol was active only when higher concentrations were applied (2–23 mg/mL). Besides realizing a high MIC value, eucalyptol treatment upregulated the expression of two genes encoding for efflux pumps and linked to *Candida* drug resistance, *CDR1* and *CDR2*, while inducing toxic effects on liver cells, leading to the conclusion that it is an unsuitable antifungal candidate. On the contrary, camphor application caused a decrease in fungal virulence in the concentration that was non-toxic, suggesting its promise as an antifungal candidate. *Candida* was also the target microorganism in the study of Wróblewska et al. [2]. The authors investigated novel strategies for the treatment of fungal skin infections, including tea tree oil (TTO) as a synergistic agent in combination with ketoconazole. The addition of TTO increased solubility of ketoconazole in a range of solvents, enhanced the penetration and retention of ketoconazole via the artificial skin membrane, and increased antifungal effectiveness towards *Candida* strains. The authors concluded that novel TTO-enriched gels are promising antifungal therapeutics due to their efficient drug release and antifungal activity. Another study examining novel therapeutic options for the treatment of *Candida* was presented by Mori et al. [3]. They studied the antifungal potential of CKR12 (a mutant fragment of the human cathelicidin peptide LL-37)-PLGA-miconazole (MCZ) micelles. The in vitro release profile of MCZ in micelles showed a time-dependent drug release at a neutral physiological pH. Novel micelles exhibited higher antifungal activity than CKR12-PLGA micelles and the MCZ solution (MICs 0.24 µM, 24.25 µM, and 24.03 µM, respectively). The mechanism of antifungal activity is based on interference with the integrity of the fungal cell wall and cell membrane. It was concluded that synergistic antifungal action can be achieved by using a combination of antimicrobial peptide fragment analogues and MCZ. Susceptibility of both fungal and bacterial pathogens to steroid-functionalized imidazolium salts, 12 of which were synthesized and characterized for the first time, was explored by Malinowska et al. [4]. Synthesized salts exhibited significant antifungal properties, with the MIC for some of the compounds lower than 0.06 µg/mL. Hemocompatibility with host cells and cytotoxicity towards fibroblasts CRL-1475 were examined, and no toxicity was observed at the antimicrobial concentration, suggesting that steroid-based imidazolium salts are promising antimicrobial compounds. Finally, an overview of novel antifungal targets and strategies is given in the review paper by Ivanov et al. [5]. The antifungal mechanisms described include interference with fungal resistance, obstruction of virulence, inhibition of fungal enzymes, metabolism, mitochondria, and cell wall integrity. Antifungal vaccines are also mentioned as a promising antifungal strategy. Numerous agents employing different antifungal mechanisms are discussed and might be more thoroughly explored in the future to develop new generations of antifungals.

Development of novel fungicides for plant protection has been the subject of numerous studies. Chen et al. [6] explored metal–organic frameworks (MOFs) as promising candidates for pesticide release carriers. Aluminum-based metal–organic framework material (NH2-Al-MIL-101) was prepared using Al3+ as the metal node and 2-aminoterephthalic acid as the organic chain. Two fungicides with different antifungal mechanisms, azoxystrobin and diniconazole, were simultaneously encapsulated into the framework. This dual fungicide delivery system exhibited sustained and pH-responsive release profiles and demonstrated a synergistic germicidal effect towards *Rhizoctonia solani*, suggesting that plant protection through co-delivery of fungicides via MOFs is a promising antifungal strategy. Yan et al. [7] explored the antifungal activity of *Humulus lupulus* ethanolic extract and its main flavonoid constituent, isoxanthohumol, against the plant pathogenic fungus *Botrytis cinerea*. The authors determined the plant extract had moderate antifungal activity, while isoxanthohumol exhibited a strong antifungal effect (EC_50_ 4.32 µg/mL). These antifungal effects were confirmed in vivo. The antifungal mechanisms of isoxanthohumol could be linked to the interferences in different aspects of metabolism as determined by transcriptome analysis. Finally, the authors concluded that isoxanthohumol has potential to be utilized for the control of phytopathogenic fungi. Yan et al. [8] also examined a novel potential fungicide for the treatment of fungal pathogen *B. cinerea,* a sulfonamide compound, *N*-(2-trifluoromethyl-4-chlorphenyl)-2-oxocyclohexyl sulfonamide (L13). L13’s mode of action includes morphological and cytological changes in *B. cinerea*. In a field study on the control of tomato gray mold, L13’s beneficial effect was more pronounced than the protective effect of commercial fungicide iprodione. The abovementioned results, along with the low toxicological effect of L13, implies that this compound has great potential to be developed as a novel fungicide. Li et al. [9] explored phenolic compounds from *Glycyrrhiza glabra* as potential antifungals for plant protection. The most promising antifungal effect was noticed for glabridin (EC_50_ 6.78 µg/mL against *Sclerotinia sclerotiorum*) and confirmed in vivo using a detached leaf assay. Its mode of antifungal action includes an increase in reactive oxygen species accumulation, the loss of mitochondrial membrane potential, and cell membrane interference. Finally, the authors concluded that glabridin demonstrates promising fungicidal properties towards *S. sclerotiorum* and might be explored as a fungicide. The study of Maniak et al. [10] provided insight into molecular mechanisms regarding inhibition of fungal laccase by studying a set of hydrazide–hydrazones against laccase from *Trametes versicolor*. The majority of synthesized molecules were strong laccase inhibitors, active in the range of *K*_I_ = 8–233 µM. Since targeted enzyme laccase enhances lignin decomposition and neutralization of the auxins and phytoalexins, which are the first line of defense against pathogens, the obtained results could indicate their promising application in the development of antifungals to reduce losses during plant cultivation and gardening. Walnut production is among the areas most negatively affected by fungal infections, precisely anthracnose disease caused by the fungus *Colletotrichum gloeosporioides*. Choub et al. [11] studied the antifungal and growth promotion activities of *Bacillus velezensis* CE 100. The crude enzyme from *B. velezensis* CE 100 induced the degradation of the *C. gloeosporioides* cell wall, inhibited spore germination, and reduced mycelial growth. This antifungal activity might be linked to the production of antifungal lytic enzymes. Inoculation of *B. velezensis* CE 100 culture broth on walnut trees drastically lowered the disease incidence; enhanced walnut root development, nutrient uptake, and chlorophyll content; and increased total biomass of the walnut trees. This study concluded that *B. velezensis* CE 100 could be used as a biocontrol agent for anthracnose disease and a walnut tree growth promotion agent. Leaf blight disease was the target of a similar study that further explored the potential application of *B. velezensis* CE 100 [12]. This disease decreases the quality of forest container seedlings such as *Quercus acutissima* seedlings. *B. velezensis* CE 100 induces cell wall lysis and hyphae deformation of *Pestalotiopsis maculans*, the causative agent of leaf blight disease. Moreover, *B. velezensis* CE 100 inoculation leads to an increase in the total nitrogen content of seedlings, an enhanced chlorophyll index in the leaves, and higher seedling biomass. Overall, *B. velezensis* CE 100 could be utilized in the eco-friendly production of high-quality forest seedlings. Besides developing novel fungicides, some authors have tried to improve the efficacy of those currently in use by developing synergistic combinations. Chitosans and copper fungicides, when combined, have a tendency to precipitate. Lemke et al. [13] examined partial hydrolysis of a chitosan polymer as a means to obtain a mixture of smaller polymers and oligomers. Such a low-molecular-weight hydrolysate could be combined with copper acetate, providing a synergistic effect. This synergistic combination could reduce copper concentration by 50% without interfering with antifungal properties, representing a step towards more sustainable agriculture practices. Toxicity is among the top reasons for the exploration and development of novel fungicides. In a review article, Yanicostas et al. [14] provide updated information regarding the toxicity of nine commonly used succinate dehydrogenase inhibitor (SDHI) fungicides towards zebrafish. SDHIs induce a range of undesirable effects in different stages of zebrafish development, including developmental toxicity, cardiovascular abnormalities, and organ damage. Glycometabolism deficit, oxidative stress, and apoptosis are critical for the toxicity of the majority of SDHIs. However, studies on toxicity are scarce, and further investigation is necessary.

Numerous studies are exploring novel agents to combat both human and plant infections caused by fungal pathogens. Despite their abundancy, fungal pathogens are still finding ways to resist antifungal treatment, making development of novel antifungals a permanent mission for scientists all across the world.

## References

[1] Ivanov M., Kannan A., Stojković D.S., Glamočlija J., Calhelha R.C., Ferreira I.C.F.R., Sanglard D., Soković M. (2021). Camphor and Eucalyptol-Anticandidal Spectrum, Antivirulence Effect, Efflux Pumps Interference and Cytotoxicity. Int. J. Mol. Sci..

[2] Wróblewska M., Szymańska E., Winnicka K. (2021). The Influence of Tea Tree Oil on Antifungal Activity and Pharmaceutical Characteristics of Pluronic^®^ F-127 Gel Formulations with Ketoconazole. Int. J. Mol. Sci..

[3] Mori T., Yoshida M., Hazekawa M., Ishibashi D., Hatanaka Y., Kakehashi R., Nakagawa M., Nagao T., Yoshii M., Kojima H. (2021). Targeted Delivery of Miconazole Employing LL37 Fragment Mutant Peptide CKR12-Poly (Lactic-Co-Glycolic) Acid Polymeric Micelles. Int. J. Mol. Sci..

[4] Malinowska M., Sawicka D., Niemirowicz-laskowska K., Wielgat P., Car H., Hauschild T., Hryniewicka A. (2021). Steroid-Functionalized Imidazolium Salts with an Extended Spectrum of Antifungal and Antibacterial Activity. Int. J. Mol. Sci..

[5] Ivanov M., Ćirić A., Stojković D. (2022). Emerging Antifungal Targets and Strategies. Int. J. Mol. Sci..

[6] Chen H., Shan Y., Cao L., Zhao P., Cao C., Li F., Huang Q. (2021). Enhanced Fungicidal Efficacy by Co-Delivery of Azoxystrobin and Diniconazole with Cauliflower-Like Metal–Organic Frameworks NH2-Al-MIL-101. Int. J. Mol. Sci..

[7] Yan Y.F., Wu T.L., Du S.S., Wu Z.R., Hu Y.M., Zhang Z.J., Zhao W.B., Yang C.J., Liu Y.Q. (2021). The antifungal mechanism of isoxanthohumol from humulus lupulus linn. Int. J. Mol. Sci..

[8] Yan X., Chen S., Sun W., Zhou X., Yang D., Yuan H., Wang D. (2022). Primary Mode of Action of the Novel Sulfonamide Fungicide against Botrytis cinerea and Field Control Effect on Tomato Gray Mold. Int. J. Mol. Sci..

[9] Li A., Zhao Z., Zhang S., Zhang Z., Shi Y. (2021). Fungicidal activity and mechanism of action of glabridin from *Glycyrrhiza glabra* L.. Int. J. Mol. Sci..

[10] Maniak H., Talma M., Giurg M. (2021). Inhibitory Potential of New Phenolic Hydrazide-Hydrazones with a Decoy Substrate Fragment towards Laccase from a Phytopathogenic Fungus: SAR and Molecular Docking Studies. Int. J. Mol. Sci..

[11] Choub V., Ajuna H.B., Won S.J., Moon J.H., Choi S.I., Maung C.E.H., Kim C.W., Ahn Y.S. (2021). Antifungal Activity of Bacillus velezensis CE 100 against Anthracnose Disease (*Colletotrichum gloeosporioides*) and Growth Promotion of Walnut (*Juglans regia* L.) Trees. Int. J. Mol. Sci..

[12] Won S.J., Moon J.H., Ajuna H.B., Choi S.I., Maung C.E.H., Lee S., Ahn Y.S. (2021). Biological Control of Leaf Blight Disease Caused by Pestalotiopsis maculans and Growth Promotion of Quercus acutissima Carruth Container Seedlings Using Bacillus velezensis CE 100. Int. J. Mol. Sci..

[13] Lemke P., Jünemann L., Moerschbacher B.M. (2022). Synergistic Antimicrobial Activities of Chitosan Mixtures and Chitosan–Copper Combinations. Int. J. Mol. Sci..

[14] Yanicostas C., Soussi-yanicostas N. (2021). SDHI Fungicide Toxicity and Associated Adverse Outcome Pathways: What Can Zebrafish Tell Us?. Int. J. Mol. Sci..

